# Supplementary material for: Household dietary diversity across regions in Ethiopia: Evidence from Ethiopian socio-economic survey data
Source: PLoS One. 2023 Apr 5;18(4):e0283496. doi: 10.1371/journal.pone.0283496 (PMC10075445; doi:10.1371/journal.pone.0283496)
Supplement: Attachment — Submitted filename: Respons to Reviewers.docx [file pone.0283496.s001.docx]

**'Response to Reviewers'**

**Journal Requirements:**

When submitting your revision, we need you to address these additional requirements.

1. Please ensure that your manuscript meets PLOS ONE's style requirements, including those for file naming. The PLOS ONE style templates can be found at <https://journals.plos.org/plosone/s/file?id=wjVg/PLOSOne_formatting_sample_main_body.pdf> and 
<https://journals.plos.org/plosone/s/file?id=ba62/PLOSOne_formatting_sample_title_authors_affiliations.pdf>

**Response**: We have attempted to follow and maintain PLOS ONE manuscript writing requirements

"PLOS requires an ORCID iD for the corresponding author in Editorial Manager on papers submitted after December 6th, 2016. Please ensure that you have an ORCID iD and that it is validated in Editorial Manager. To do this, go to ‘Update my Information’ (in the upper left-hand corner of the main menu), and click on the Fetch/Validate link next to the ORCID field. This will take you to the ORCID site and allow you to create a new iD or authenticate a pre-existing iD in Editorial Manager. Please see the following video for instructions on linking an ORCID iD to your Editorial Manager account: <https://www.youtube.com/watch?v=_xcclfuvtxQ>"

[Note: HTML markup is below. Please do not edit.]

**Response**: All the authors have ORCID ID

**Reviewers' comments:**

Reviewer's Responses to Questions

**Comments to the Author**

1. Is the manuscript technically sound, and do the data support the conclusions?

The manuscript must describe a technically sound piece of scientific research with data that supports the conclusions. Experiments must have been conducted rigorously, with appropriate controls, replication, and sample sizes. The conclusions must be drawn appropriately based on the data presented.

Reviewer #1: No

**Response**: The conclusion section is revisited as per the reviewer’s suggestion (see the comment track in the conclusion section for the change). However, the study is conducted under a social science setting in which experimental design with controls is not feasible. The sample size for the study is big enough to represent the rural household population as we have used a data set from a national survey. The approach we followed is to control variables that explain dietary diversity of households by accounting for them through regression estimation so that we avoid omitted variable bias. The type of regression suitable for estimating is the ordered logit model which estimates the contributions of each variables (in our case confounding variables) that determine the probability of falling into a particular dietary diversity category.

2. Has the statistical analysis been performed appropriately and rigorously?

Reviewer #1: No

Response: Based on this, we have done the following

- Abstract: Under result paragraph, we have included AOR with 95% CI based on the comments.
- Additional explanations are included under “variable and method of data analysis” sections (Refer to 2.2 and 2.3 sub headings). The explanations in the methodology section indicates that we have used appropriate and rigorous methods, analysis and interpretation as deemed appropriate as per the nature of the variables and objectives of the study.

3. Have the authors made all data underlying the findings in their manuscript fully available?

The [PLOS Data policy](http://www.plosone.org/static/policies.action#sharing) requires authors to make all data underlying the findings described in their manuscript fully available without restriction, with rare exception (please refer to the Data Availability Statement in the manuscript PDF file). The data should be provided as part of the manuscript or its supporting information, or deposited to a public repository. For example, in addition to summary statistics, the data points behind means, medians and variance measures should be available. If there are restrictions on publicly sharing data—e.g. participant privacy or use of data from a third party—those must be specified.

Reviewer #1: Yes

4. Is the manuscript presented in an intelligible fashion and written in standard English?

PLOS ONE does not copyedit accepted manuscripts, so the language in submitted articles must be clear, correct, and unambiguous. Any typographical or grammatical errors should be corrected at revision, so please note any specific errors here.

Reviewer #1: Yes :

**Additional**: We have taken the leverage to further edit the language and improve the presentations of the technical findings so as to improve its technical soundness.

5. Review Comments to the Author

Please use the space provided to explain your answers to the questions above. You may also include additional comments for the author, including concerns about dual publication, research ethics, or publication ethics. (Please upload your review as an attachment if it exceeds 20,000 characters)

Reviewer #1: Even if this paper addressed important area of researchable topic with enough sample size, the following points should be addressed before publishing this paper.

Line1: Title of the study is that equivalent to household dietary diversity? If not what is there difference?

**Response:** The title is to refer to household dietary diversity. We were trying to be more specific by including ‘Rural’ in the title as the study considered only those households living in rural areas. As per the comment of the reviewer, we removed the term ‘rural’ in the title, and mentioned the focus of the study under ‘’the scope’’ section of the manuscript.

Line19: Result: better to start by answering your objectives. Additionally include AOR with 95% CI to the predictor variables.

**Response**: As per the comment, AOR for each predictor variable is included in the result sections of the abstract

Line 114: Variables: Which one is the validated tool in Ethiopia to measure household food security from the four methods you listed? And why you preferred HDDS? Again this part should be discussed in the introduction session.

**Response**: Commonly food security studies in Ethiopia adopted individual calorie intake/ household caloric consumption to measure food security status and its determinants. In this case, the focus is on diet quality rather than total energy intake: The DDS takes into account the diversity of foods consumed, which can provide a more nuanced understanding of diet quality than the calorie consumption method, which only considers total energy intake. The measurement indicator (i.e dietary diversity) is also scientifically proved by various scholars as shown in the manuscript. See the response in the method section of manuscript .

Line 129: Table 1: I don’t understand the importance of this table. Better if you remove it.

**Response**: the comment is well taken. The table is replaced with texts.

Line 132: wealth index analysis method should be described under data analysis sub heading.

**Response**: Detail description of the procedure to construct wealth index is presented in the method section of the manuscript.

139-172: Data analysis method is not well explained at all. You need to write what you did in steps. No need of explaining the formula for the model you used.

**Response**: The section is elaborated based on the comments.

Line196: Table 2: the title of the table should be self-explanatory

**Response**: Modified as suggested

Line209: which statistical method you used to check the difference in HDDS across regions? It is not explained.

**Response**: Chi square analysis was done to show dietary diversity across regions. This is presented in the last column of table 2 of the original manuscript. We explained in the revised version the statistical tool we used to examine DDS across regions

Line270 Table 4: see the above comment.

**Response**: We feel that removing table 4 (in the revised version, table 3) will reduce the technical soundness/scientific status of the manuscript, and suggest to maintain it.

Line 247: Which model you applied to identify independent factors associated with HDDS? Have controlled confounders? Why you didn’t use different subheadings for result and discussion?

**Responses**:

- Which model you applied to identify independent factors associated with HDDS?

We applied the ordered logit model to identify independent factors associated with DDS. It is explained under the ‘Method’ section of the manuscript.

- Have controlled confounders?

In the method section of the manuscript, the tools we adopted to identify/control confounding factors are explained. We used Variance Inflation Factor (VIF) for continuous and Contingency Coefficient (CC) for discrete variables to identify confounding variables, and if exist to exclude them from the ordered logit model.

- Why you didn’t use different subheadings for result and discussion?

We feel providing discussion with results in the same heading gives better opportunity to get information at the same place. It is a matter of convention/style. If the reviewer still feels that this is the best approach, we are willing to do it. But, we leave it under same sub-heading for now.

6. PLOS authors have the option to publish the peer review history of their article ([what does this mean?](https://journals.plos.org/plosone/s/editorial-and-peer-review-process#loc-peer-review-history)). If published, this will include your full peer review and any attached files.


If you choose “no”, your identity will remain anonymous but your review may still be made public.


**Do you want your identity to be public for this peer review?** For information about this choice, including consent withdrawal, please see our [Privacy Policy](https://www.plos.org/privacy-policy).

Reviewer #1: No

[NOTE: If reviewer comments were submitted as an attachment file, they will be attached to this email and accessible via the submission site. Please log into your account, locate the manuscript record, and check for the action link "View Attachments". If this link does not appear, there are no attachment files.]

While revising your submission, please upload your figure files to the Preflight Analysis and Conversion Engine (PACE) digital diagnostic tool, <https://pacev2.apexcovantage.com/>. PACE helps ensure that figures meet PLOS requirements. To use PACE, you must first register as a user. Registration is free. Then, login and navigate to the UPLOAD tab, where you will find detailed instructions on how to use the tool. If you encounter any issues or have any questions when using PACE, please email PLOS at [figures@plos.org](mailto:figures@plos.org). Please note that Supporting Information files do not need this step.

*In compliance with data protection regulations, you may request that we remove your personal registration details at any time.* [*(Remove my information/details)*](https://www.editorialmanager.com/pone/login.asp?a=r)*. Please contact the publication office if you have any questions.*
