# Supplementary material for: Household dietary diversity across regions in Ethiopia: Evidence from Ethiopian socio-economic survey data
Source: PLoS One. 2023 Apr 5;18(4):e0283496. doi: 10.1371/journal.pone.0283496 (PMC10075445; doi:10.1371/journal.pone.0283496)
Supplement: Attachment — Submitted filename: Respons to Reviewers.docx [file pone.0283496.s002.docx]

'Response to Reviewers'

Additional Editor Comments:

**Comment: In abstract 95% CI is not indicated??**

Response: In the abstract, the CI was mentioned/included without mentioning 95%. This time following the comment, 95% is added to explain more at what level the CI was generated.

**Comment: In table 1 Mean ([stand.dev/range](http://stand.dev/range)) ...mean can be reported with standard deviation not with range.**

Response: Range was deleted from the column title, and standard deviation was calculated and included for ‘’Household size’’ variable.
